# Willingness to use HIV self‐test kits and willingness to pay among urban antenatal clients in Cote d’Ivoire and Tanzania: a cross‐sectional study

**DOI:** 10.1111/tmi.13456

**Published:** 2020-07-29

**Authors:** Kim Ashburn, Gretchen Antelman, Marc Kouadio N'Goran, Ola Jahanpour, Aida Yemaneberhan, Bernard N'Guessan Kouakou, Erin Kazemi, Malia Duffy, Pongathie Adama, Deborah Kajoka, Alexandra Coombs, Josef Tayag, David Sullivan, Alex Vrazo

**Affiliations:** ^1^ Elizabeth Glaser Pediatric AIDS Foundation Washington DC USA; ^2^ Elizabeth Glaser Pediatric AIDS Foundation Dar‐es‐Salaam Tanzania; ^3^ Elizabeth Glaser Pediatric AIDS Foundation Adibjan Cote d'Ivoire; ^4^ JSI Research and Training Institute, Inc Arlington VA USA; ^5^ Ministry of Health Abdijan Cote d'Ivoire; ^6^ Ministry of Community Development, Gender, Elderly and Children Dar es Salaam Tanzania; ^7^ United States Agency for International Development Washington DC USA

**Keywords:** cross‐sectional survey, HIV self‐test kit, willingness to pay, acceptability, antenatal clients, Tanzania, Cote d’Ivoire, enquête transversale, kit d'auto‐dépistage du VIH, volonté de payer, acceptabilité, patientes prénatales, Tanzanie, Côte d'Ivoire

## Abstract

**Objectives:**

To generate evidence on willingness to use HIV self‐test kits and willingness to pay among antenatal care clients in public and private facilities in Cote d’Ivoire and Tanzania.

**Methods:**

Cross‐sectional survey data were collected from 414 clients recruited from 35 high‐volume facilities in Cote d’Ivoire and from 385 clients in 33 high‐volume facilities in Tanzania. Surveys covered willingness to use HIV self‐test kits, prices clients were willing to pay, advantages and disadvantages and views on specific qualities of HIV self‐tests. Market data on availability of proxy self‐testing products (e.g. pregnancy and malaria tests) and attitudes of pharmacists towards HIV self‐test kits were collected from 51 pharmacies in Cote d’Ivoire and 59 in Tanzania.

**Results:**

Willingness to use HIV self‐test kits was 65% in Cote d’Ivoire and 69% in Tanzania. Median ideal prices women would pay ranged from USD 1.77 in Cote d’Ivoire to USD 0.87 in Tanzania. Proxy self‐test kits were available in pharmacies, and interest was high in stocking HIV self‐test kits.

**Conclusions:**

Implications for national HIV self‐test policy and planning include keeping prices low, providing psychological and HIV counselling, and ensuring linkage to HIV care and treatment services. Private pharmacies will play a key role in providing access to HIV self‐test kits.

## Abstract (fr)

**Objectifs:**

Générer des données sur la volonté d'utiliser des kits d'auto‐dépistage du VIH et la volonté de payer chez les clients des soins prénatals dans les établissements publics et privés en Côte d'Ivoire et en Tanzanie.

**Méthodes:**

Les données de l'enquête transversale ont été recueillies auprès de 414 patientes enrôlées dans 35 établissements de santé à volume élevé en Côte d'Ivoire et de 385 patientes dans 33 établissements de santé à volume élevé en Tanzanie. Les enquêtes ont porté sur la volonté d'utiliser des kits d'auto‐dépistage du VIH, les prix que les clients étaient prêts à payer, les avantages et les inconvénients et les opinions sur les qualités spécifiques des auto‐tests du VIH. Les données du marché sur la disponibilité des proxyse des produits d'auto‐test (par exemple, les tests de grossesse et du paludisme) et les attitudes des pharmaciens envers les kits d'auto‐test du VIH ont été collectées dans 51 pharmacies en Côte d'Ivoire et 59 en Tanzanie.

**Résultats:**

La volonté d'utiliser des kits d'auto‐test VIH était de 65% en Côte d'Ivoire et de 69% en Tanzanie. Le prix médian idéal que les femmes paieraient allait de 1,77 USD en Côte d'Ivoire à 0,87 USD en Tanzanie. Des proxys de kits d'auto‐test étaient disponibles dans les pharmacies et l'intérêt pour le stockage des kits d'auto‐test du VIH était élevé.

**Conclusions:**

Les implications pour la politique et la planification nationales d'auto‐test du VIH incluent le maintien de prix bas, la fourniture de conseils psychologiques et sur le VIH, et la garantie d'un lien avec les services de soins et de traitement du VIH. Les pharmacies privées joueront un rôle clé en donnant accès aux kits d'autotest du VIH.

## Introduction

HIV testing is a prerequisite to accessing HIV care and treatment services, yet in some regions of sub‐Saharan Africa, only 48% of individuals living with HIV know their status [1]. Disparities between women and men exist across the region, with lower testing rates among men [1]. This disparity is due in part to women accessing HIV testing in antenatal care (ANC) and other reproductive healthcare settings, and men having fewer opportunities to interact with the healthcare system for HIV testing services [2]. HIV testing barriers among couples and individuals are widely documented and include stigma and discrimination, challenges at facilities such as long waiting times, not knowing the benefits of HIV testing and economic and opportunity costs [3, 4, 5]. HIV counselling and testing are associated with reduced HIV risk including reduction in multiple partners and increased condom use, particularly among people diagnosed with HIV [6]. As outlined in UNAIDS 90‐90‐90 goals, knowing one's HIV status is the first step in controlling the HIV epidemic [7]. New and innovative ways to expand access to HIV testing are required to achieve this important goal.

Recent momentum towards country‐level regulatory approval and targeted distribution of HIV self‐test kits (HIVSTK) as an alternative to facility‐based HIV testing services offers a new and potentially important strategy to address testing gaps [8]. HIV self‐testing (HIVST) allows an individual to collect their own oral fluid or blood specimen, conduct an HIV test in a private setting and independently interpret the result [9]. HIV self‐testing has the potential to address key testing barriers, including confidentiality and privacy concerns, stigma and discrimination, accessibility and wait time for results [10]. Evidence from multiple pilot studies in sub‐Saharan Africa demonstrates that HIV self‐testing is highly feasible, acceptable and accurate across populations including ANC clients in Malawi [11], sex workers in Uganda [12] and MSM in Nigeria [13]. HIVST can lead to earlier diagnosis if successfully integrated with education and support to ensure high rates of return for confirmatory testing [14] and is cost‐effective in high HIV prevalence settings [15]. Data from a randomised controlled trial in Kenya showed that distribution of HIVSTK to ANC clients increased uptake of male partner testing [16]. Also in Kenya, distribution of HIVSTK among sex workers had positive effects on promoting safer sex practices [17]. Studies in Kenya, South Africa, Tanzania and Zambia have shown a potential willingness to pay (WTP) for HIVSTK [18, 19, 20, 21, 22].

In 2016, the WHO published guidelines for HIVST [9], and many countries in sub‐Saharan Africa have included HIV self‐testing in strategic plans. Establishing affordable prices and safe and confidential settings in which to offer self‐testing, including in the private sector, for equitable access are challenging issues. At the time of data collection for this study, Cote d’Ivoire and Tanzania were piloting HIVSTK, and Tanzania was developing a national HIVST policy. Few studies have collected data on willingness to pay in sub‐Saharan Africa, providing little evidence to inform policy planning. The aim of this study was to generate evidence on willingness to use HIVST among ANC clients, and specific price points ANC clients are willing to pay for HIVSTK in four urban settings in Cote d’Ivoire and Tanzania.

## Methods

### Study design

This cross‐sectional study employed mixed methods to gather data on current markets and willingness to use HIVSTK and prices ANC clients were willing to pay for HIVSTK. Methods applied included mapping availability and pricing of self‐test proxy products. HIVSTK are not yet publically available in study countries, thus proxy products in the category of self‐test kits (e.g. malaria self‐test kits, pregnancy self‐test kits) were used to explore the provision of products similar to HIVSTK in the study areas. In addition, quantitative structured surveys were used to describe the willingness to use HIVSTK among a cross‐section of female consumers of ANC services and potential providers and suppliers, respectively.

### Setting

HIV self‐testing is new, with access most likely to be initiated in larger urban areas. For this reason, the study setting focused on four urban areas in each country. We selected Cote d’Ivoire and Tanzania as countries interested in piloting HIVSTK and at the time of the study were exploring ways to make the kits available. In Cote d’Ivoire, Abidjan and Bouaké districts, and in Tanzania, Dodoma and Arusha city districts were selected. In each study area, public and private health facilities and pharmacies were purposively selected for inclusion in the study. Private sector facilities included for‐profit facilities and faith‐based facilities; public sector facilities were government‐supported and managed facilities. Data were collected in 2018, from May 17th to June 9th in Tanzania and from June 4th to 22nd in Cote d’Ivoire.

The Health Facility National Database was used to select facilities for client recruitment in Cote d’Ivoire. From a list of 255 health facilities (public and private), 35 facilities were identified as meeting the criterion of high volume and selected for client survey recruitment. In Tanzania, the Health Facility Register listed a total of 182 facilities, of which ANC clients were recruited from 33 high‐volume facilities.

For the market assessment, pharmacy shops located near the selected ANC facilities were selected, comprising 51 public and private pharmacies in Cote d’Ivoire and 59 private pharmacies in Tanzania.

### Study population

The study population consisted of ANC clients and pharmacists. Convenience sampling was used as the most feasible option to facilitate rapid recruitment ANC clients for structured surveys during their routine clinic visits. Eligibility criteria for participation in the structured surveys included being a current ANC client in a private or public study facility and age 18 years or older. All participants provided written informed consent prior to interview. Pharmacists who offer HIV proxy products (pregnancy and/or malaria self‐tests) were recruited during visits to purposively selected pharmacies located in the service areas of study facilities. A sample size estimate of 192 ANC clients per district, per country, was based on a conservative estimate of 50% of ANC clients being willing to use HIVSTK and allowed an estimate within a margin of error of 5% of the population proportion.

### Data collection procedures and data analysis

All mapping and cross‐sectional survey data were collected electronically using handheld tablets by trained data collectors. All data collection forms were standardised across the study countries with range and value checks programmed into the data collection forms to reduce data entry errors. Measures of willingness to pay were adapted from the literature on stated preferences using what price the respondent considers normal or ideal, too expensive and too cheap. The survey began with a short description of an oral HIVSTK. Data collection forms were developed in English and translated into French and Swahili for data collection in Cote d’Ivoire and Tanzania, respectively.

Analysis of market mapping and ANC client surveys was performed using Stata version 15.0. Descriptive statistics were used to summarise categorical (proportions, frequencies) and continuous variables (means, medians, standard deviations and interquartile ranges). Willingness to use an HIVSTK was estimated as the proportion of ANC clients surveyed who said they would ‘surely use’ an HIVSTK in the future. Logistic regression models (unadjusted and adjusted) were used to identify factors associated with willingness to use HIVSTK in the future. Tests of independence were conducted using chi square to compare key outcomes across districts. The potential for HIVSTK access and use was assessed by triangulating data from market mapping and structured surveys among ANC clients.

### Ethics

National and US‐based institutional review boards (IRB) approved this protocol including the National Committee for Research Ethics in Cote d’Ivoire; the National Institute for Medical Research IRB in Tanzania, and the Advarra IRB in the United States.

## Results

### Market availability of HIVST proxy products

Results of assessing market availability of HIVST proxy products are outlined in Table 1. A total of 33 private pharmacies were visited in Cote d’Ivoire and 59 in Tanzania. Proxy product self‐test kits were widely available, with pregnancy tests being the most common type of self‐test kits in pharmacies visited, 61% in Cote d’Ivoire and 83% in Tanzania. Over 70% of pharmacies supplied prescription medication across study sites. Clinical consultations were available in 94% of pharmacies in Cote d’Ivoire and 54% in Tanzania. Condoms were available in 97% and 81% of pharmacies in Cote d’Ivoire and Tanzania, respectively.

**Table 1:** Market availability of products, services and proxy HIVSTK at private pharmacies

Products offered	Cote d’Ivoire *N* = 33	Tanzania *N* = 59
	*n* (%)	*n* (%)
Medicine prescribed by clinician	33 (100.0)	50 (84.7)
Over‐the‐counter medicines	32 (97.0)	39 (66.1)
Self‐test kits	33 (100.0)	54 (91.5)
Lab testing	15 (45.5)	13 (22.0)
Medical supplies/ consumables	29 (87.9)	18 (30.5)
Clinical consultation	31 (93.9)	32 (54.2)
Beauty products (soap, lotions, vitamins)	33 (100.0)	29 (49.2)
Condoms	32 (97.0)	48 (81.4)
Other	3 (9.1)	4 (6.8)
Types of self‐test kits offered		
Pregnancy	20 (60.6)	49 (90.7)
Glycaemia	18 (54.5)	22 (40.7)
Malaria	16 (48.5)	6 (11.1)

Pharmacists had positive perceptions of HIVSTK. Nearly 91% of pharmacists in Cote d’Ivoire said they would be interested in stocking HIVSTK and 78% said the same in Tanzania (data not shown). Table 2 shows pharmacist‐reported stock outs and supply chain challenges. Stock outs were common, occurring ‘sometimes’ or ‘often’ in 86% of sites in Cote d’Ivoire and 70% in Tanzania. Transportation was the most frequently mentioned supply chain issue in Tanzania (38%). In Cote d’Ivoire, receiving less than ordered (69%) and receiving different than ordered (67%) were the most often cited challenges.

**Table 2:** Stock outs and supply chain challenges among all pharmacy shops

Supply chain or other challenge	Cote d’Ivoire Pharmacies *N* = 51 *n* (%)	Tanzania Pharmacies (*N* = 59) *n* (%)
Stock outs sometimes or often	44 (86.3)	41 (69.5)
Transportation	7 (13.7)	22 (37.3)
Sourcing supply	10 (19.6)	16 (27.1)
Received less than ordered	35 (68.6)	18 (30.5)
Received different than ordered	34 (66.7)	11 (18.6)
Inventory control	8 (15.7)	3 (5.1)
Storage	7 (13.7)	6 (10.2)
High cost, price fluctuation	N/A	22 (37.3)
Multiple brands make clients choosy	N/A	3 (5.1)
Clients not tested/diagnosed	N/A	3 (5.1)
Not registered/registration changes	N/A	2 (3.4)

### ANC client surveys

A total of 799 ANC clients were surveyed; 414 in Cote d’Ivoire and 385 in Tanzania. Table 3 shows demographic characteristics. In both Cote d’Ivoire and Tanzania, the median age among ANC clients was 25 years, about two‐thirds reported earning their own money (66% and 68%, respectively), and only a minority (15% and 19%, respectively) had health insurance.

**Table 3:** Demographic characteristics for ANC clients

Variables	Cote d’Ivoire *N* = 414 *n* (%)	Tanzania *N* = 385 *n* (%)	*P*‐value*
Age			0.003
18–24 years	139 (33.6)	174 (45.2)	
25–34 years	216 (52.2)	169 (44.0)	
35+	59 (14.3)	42 (11.0)	
Education			<0.001
No school	94 (22.7)	12 (3.1)	
Primary	119 (28.7)	155 (40.3)	
Secondary	116 (28.0)	164 (43.9)	
Post‐secondary	85 (20.5)	54 (10.9)	
Socioeconomic status			<0.001
Low	42 (10.1)	112 (29.1)	
Medium	265 (64.0)	186 (48.3)	
High	107 (29.9)	87 (22.6)	
Main source of income in past year			<0.001
Farming	8 (1.9)	38 (9.9)	
Formally employed	124 (30.0)	77 (20.0)	
Informally employed	128 (30.9)	57 (14.8)	
Self‐employed	117 (28.3)	202 (52.5)	
Other	37 (8.9)	11 (2.9)	
Earn own money			0.43
Yes	272 (65.7)	263 (68.3)	
No	142 (34.3)	122 (31.7)	
Who makes decisions about how to use money?			<0.001
I alone decide	200 (48.3)	125 (47.5)	
My partner/husband	52 (12.6)	23 (8.8)	
My partner/husband and I decide	123 (29.7)	111 (42.2)	
Another male relative decides	15 (3.6)	0 (0.0)	
Other	24 (5.8)	4 (1.5)	
Have health insurance			0.19
Yes	63 (15.2)	72 (18.7)	
No	351 (84.8)	313 (81.3)	

* Pearson's Chi square test or Fischer's exact test used for small values.

There were notable differences between the two country samples; however, in education, source of income, and socioeconomic status. In Cote d’Ivoire, more than half of women had only primary (29%) or no education (23%), vs in Tanzania where most women had at least primary (40%) or secondary (44%) education and very few (3%) reported no formal schooling (*P* < 0.001). Socioeconomic status (SES) was measured using a household assets index. There were significant differences in SES with 64% vs 48% medium SES and 10% vs 29% low income in Cote d’Ivoire and Tanzania, respectively.

In Cote d’Ivoire, nearly all women had some experience using a self‐test kit, with 87% having used a pregnancy test. Women paid a median price of 1000 CFA francs (CFA) ($1.73 USD) for any self‐test kit they used in the past. In Tanzania, 67% of women reported ever using a self‐test kit for pregnancy, less than 5% had used other types of self‐test kits such as for malaria or blood sugar. The median price paid in the past for any self‐test kit was 1000 Tanzania shillings (TSH) ($0.44 USD) and most paid less than 1500 TSH ($0.66 USD).

### Acceptability of HIVST kits among ANC clients

In both Cote d’Ivoire and Tanzania, acceptability of HIVSTK was very high among all women (Table 4). A total of 90% and 86%, respectively, said they would ‘surely use’ or ‘maybe use’ an HIVSTK in the future, and 64% reported HIVSTK as an idea that they ‘like it a lot’. In Cote d’Ivoire, 18% of women said they would feel ‘somewhat uncomfortable’ or ‘very uncomfortable’ taking the HIV self‐test alone without a health provider or counsellor, and 22% would be ‘somewhat’ or ‘very uncomfortable’ bringing the HIVSTK home to share with their partner. In Tanzania, women were almost twice as likely (32%) to report discomfort testing without a health provider, but reported similar levels of discomfort in bringing an HIVSTK to their partner at home (17% very or somewhat uncomfortable).

**Table 4:** Willingness to use HIVSTK, attitudes and preferences

Variables	Cote d’Ivoire *N* = 414 *n* (%)	Tanzania *N* = 385 *n* (%)	*P*‐value*
Do you think you would want to use an HIV self‐test kit in the future?			<0.001
Would surely use	269 (65.0)	264 (68.6)	
Maybe use	105 (25.4)	64 (16.7)	
Probably not use	15 (3.6)	8 (2.1)	
Would surely not use	15 (3.6)	43 (11.2)	
Don't know	10 (2.4)	6 (1.6)	
How much do you like the idea of the HIV self‐test kit?			<0.001
Like it a lot	265 (64.0)	248 (64.4)	
Like it a little	126 (30.4)	96 (24.9)	
Dislike	16 (3.7)	35 (9.1)	
Don't know	7 (1.7)	6 (1.6)	
How comfortable would you feel about taking the HIV self‐ test kit on your own, without a health provider or counsellor?			<0.001
Very comfortable	202 (48.8)	172 (44.7)	
Somewhat comfortable	126 (30.4)	90 (23.4)	
Somewhat uncomfortable	54 (13.0)	39 (10.1)	
Very uncomfortable	19 (4.6)	84 (21.8)	
Don’t know	13 (3.1)	0 (0.0)	
How comfortable would you feel about bringing an HIV self‐test kit home to share with your partner/husband?			<0.001
Very comfortable	168 (40.6)	259 (67.3)	
Somewhat comfortable	141 (34.1)	63 (16.4)	
Somewhat uncomfortable	55 (13.3)	10 (2.6)	
Very uncomfortable	35 (8.5)	53 (13.8)	
Don't know	15 (3.6)	0 (0.0)	
How important: accuracy of the test			<0.001
Very important	256 (61.8)	275 (71.4)	
Somewhat important	141 (34.1)	73 (19.0)	
Not important	17 (4.1)	37 (9.6)	
How important: the test is easy to use			0.649
Very important	295 (71.3)	275 (71.4)	
Somewhat important	100 (24.2)	75 (19.5)	
Not important	19 (4.6)	35 (9.1)	
How important: confidentiality of taking a test alone			<0.001
Very important	262 (63.3)	189 (49.1)	
Somewhat important	130 (31.4)	111 (28.8)	
Not important	22 (5.3)	85 (22.1)	

* Pearson's Chi Square Test or Fischer's Exact Test used for small values.

Attitudes and preferences about HIVSTK were significantly different across Cote d’Ivoire and Tanzania. A higher proportion of women in Tanzania than in Cote d’Ivoire said accuracy of the test was very important, 71% vs 62%, respectively (*P* < 0.001). There was agreement on the importance of ease of use with nearly three‐quarters (71%) saying this was very important in both countries. As far as confidentiality of the test, a higher proportion of women in Cote d’Ivoire valued this more with 63% saying confidentiality was ‘very important’ vs (49% in Tanzania (*P* < 0.001). Attitudes were mostly positive towards using an HIVSTK in the future with at least 65% of women across the study countries saying they would sure use an HIVSTK in the future. However, more women in Tanzania said they would surely not use an HIVSTK in the future, (11%) that they disliked the idea of the HIVSTK and that they would feel very uncomfortable testing alone (22%) or sharing an HIVSTK with their partner (14%).

### Advantages and disadvantages of HIVST kits

**Figure 1:**
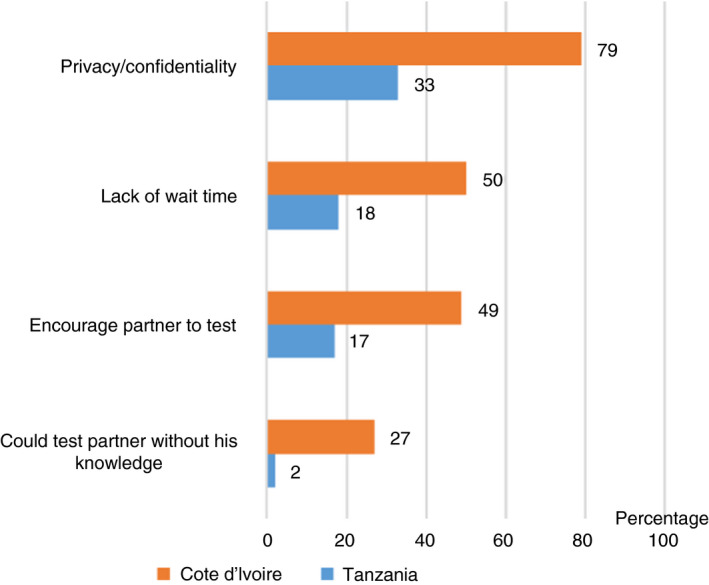
Main advantages of HIVST kits.

Figure 1 outlines women's views on the main advantages and disadvantages of HIVST. In Cote d’Ivoire, women perceived the main advantage of using HIVST was to have privacy and confidentiality in testing alone (79%). Half (50%) of women said the main advantage was the personal convenience of no waiting time. Nearly half (49%) of women said that the main advantage was to encourage their partner to test, and 27% said the main advantage is that they could test their partner without his knowledge. In Tanzania, approximately one third of women said privacy and confidentiality were the main advantages of HIVST and 18% said the lack of waiting time is the main advantage; 17% of women saw the main advantage of HIVST was that it could encourage their partner to test.

**Figure 2:**
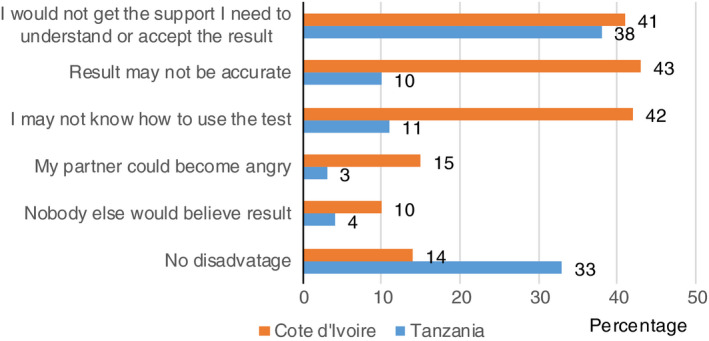
Main disadvantages of HIVST kits.

Across all study sites, one of the main disadvantages women cited was not ‘getting the support I need to understand or accept the result’, in 170 (41%) women in Cote d’Ivoire and 146 (38%) women in Tanzania. In Cote d’Ivoire, other main disadvantages women mentioned were concerns about using the test correctly and getting a result that others would consider valid. For 178 (43%) women, the main disadvantage of HIVST was that the test result may not be accurate. In 174 (42%) cases, the main disadvantage was that they may not know how to use the test kit, and for 41 (10%) that ‘nobody else would believe or trust the result’. In Tanzania, accuracy of the test and ease of using the test were the main disadvantages for only 39 (10%) and 42 (11%) of women. Few women said the main disadvantage is their partner becoming angry (4%) or someone else not believing the test result (3%) (Figure 2).

### Willingness to pay for HIVSTK

Table 5 outlines prices women were willing to pay for HIVSTK. In Cote d’Ivoire, the median ideal price women would pay for an HIVSTK was 1000 CFA ($1.77 USD). This is the same median ideal price women paid for self‐test kits used in the past. Among those women who said accuracy of the test was an important quality, the median price these women were willing to pay for a more accurate test compared to accessing a free test was not different, at 1000 CFA ($1.77 USD). In Tanzania, the median ideal price women were willing to pay for an HIVSTK was TSH 2,000 ($0.86 USD) and most women said the ideal price would be less than TSH 3500 ($1.52 USD). The majority of women said TSH 10 000 ($4.33 USD) would be too expensive for an HIVSTK. Among women who said accuracy of the test was important to them, the median price they would pay for an HIVSTK that was more accurate was TSH 5,000 ($2.16 USD).

**Table 5:** Price points willing to pay for HIVSTK

	Price in local currency* (median, Q3–Q1)		Price in USD (median, Q3–Q1)	
	Cote d’Ivoire	Tanzania	Cote d’Ivoire	Tanzania
What price would you consider to be normal/ideal for a HIV self‐test kit? (median, Q3–Q1)	1000, 1000–500	2000, 3500–1000	$1.77 (1.77–0.89)	$0.88 (0.66–0.44)
What price do you consider inexpensive? (median, Q3–Q1)	500, 500–250	1000, 2000–1000	$0.88 (0.88–0.44)	$0.44 (0.88–0.44)
What price do you consider expensive? (median, Q3–Q1)	2000, 5000–1000	5000, 10 000–5000	$3.53 (8.83–1.77)	$2.22 (4.44–2.22)
What price would you consider the HIV self‐test kit to be too expensive? (median, Q3–Q1)	5000, 6000–2000	10 000, 10 000–30 000	$8.83 (10.60–3.53)	$4.44 (4.44 –13.33)
For those who said the accuracy of the test was important; if the HIV self‐test kit was more accurate compared to free HIV testing at this clinic, what price would you be willing to pay for the HIV self‐test kit? (median, Q3–Q1)	1,000, 1,000–500	5,000, 5,000–1,500	$1.77 (1.77–0.89)	$2.22 (2.22–0.67)

* Currency exchange as of August 25, 2018: 565.9 CFA = 1 USD; 2,250 TSH = I USD.

### Factors associated with willingness to use HIVST

Regression modelling was used to identify factors associated with willingness to use HIVST. Results are shown in Tables 6 and 7. In unadjusted models in Cote d’Ivoire, factors associated with willingness to use HIVSTK in the future were women with higher levels of education, higher levels of SES, having health insurance, earn own income, a history of using self‐tests, women using facilities managed by faith‐based organisations, and women who felt ‘very comfortable’ testing on their own or sharing the HIVSTK with their male partner.

**Table 6:** Factors associated with willingness to use HIVSTK in Cote d’Ivoire

Variable	Unadjusted		
	Risk ratio	95% CI	*P*‐value
Age	1.01	1.00–1.02	0.12
Education			
No school			
Primary	1.52	1.18–1.97	0.001
Secondary	1.60	1.24–2.10	<0.001
Post‐secondary	1.66	1.30–2.14	<0.001
Health insurance (yes)	1.40	1.22–1.60	<0.001
SES			
Low (ref)			
Medium	1.33	1.0–1.90	0.09
High	1.60	1.13–2.22	0.007
Earn own income (yes)	1.30	1.10–1.54	0.002
History of self‐test (yes)	1.47	1.08–2.00	0.02
Type of facility			
Public (ref)			
Private	1.21	1.00–1.53	0.11.
Faith‐based	1.19	1.00–1.43	0.05
Comfort in taking test on your own (very comfortable)	1.89	1.61–2.21	<0.001
Comfort in sharing test with partner (very comfortable)	1.81	1.58–2.01	<0.001

**Table 7:** Factors associated with willingness to use HIVSTK in Tanzania

Variable	Unadjusted		
	Risk ratio	95% CI	p‐value
Age	1.00	0.99–1.01	0.91
Education			
No school (ref)	1.00		
Primary	0.81	0.61–1.06	0.12
Secondary	0.83	0.64–1.10	0.19
Post‐secondary	0.80	0.58–1.10	0.17
Health insurance (yes)	0.92	0.76–1.11	0.37
SES			
Low (ref)	1.00		
Medium	0.94	0.81–1.10	0.44
High	0.95	0.79–1.14	0.59
Earn own income (yes)	1.03	0.89–1.19	0.70
History of self‐test (yes)	1.18	1.00–1.38	0.05
Type of facility			
Public (ref)	1.00		
Private	1.21	1.02–1.43	0.03
Faith‐based	1.13	0.97–1.33	0.12
Comfort in taking test on your own (very comfortable)	1.42	1.2–1.62	<0.001
Comfort in sharing test with partner (very comfortable)	1.77	1.44–2.16	<0.001

Factors associated with willingness to use HIVSTK in the future in Tanzania are shown in Table 7. Factors include history of self‐test, accessing services in a private facility, women who were ‘very comfortable’ in using an HIVSTK on her own and women who were ‘very comfortable’ in sharing an HIVSTK with her partner.

## Discussion

We found a high proportion of pregnant women attending ANC were willing to use HIVSTK in the future, with the price they were willing to pay for HIVSTK similar to that for pregnancy self‐test kits. These findings add to the growing literature on the acceptability of HIVST as an alternative to traditional HIV testing and counselling services (HTS), and to the scant literature on prices, potential consumers are willing to pay for HIVSTK. Proxy products were readily available in pharmacies, and private sector interest in supplying HIVSTK was high.

Pricing will be a crucial aspect of ensuring access to ordinary consumers. Women in our study were willing to pay an ideal median price of $1.77 USD in Cote d’Ivoire and $0.87 USD in Tanzania for an HIVSTK. Other studies on willingness to pay for HIVSTK, including in Tanzania, similarly demonstrate preferences for low‐cost HIVSTK [14, 15, 16, 17, 18]. This is consistent with the current policies mandating that HIV testing services (and antenatal care) be provided free of charge in Cote d’Ivoire and Tanzania in both public and private facilities. Thus, a majority of health‐seeking clients may expect free self‐testing services from public facilities and only tolerate low‐cost testing options from private facilities or pharmacies and the introduction of HIVST services will likely require differential strategies depending on the target population and type of facility. Still, data from Cote d’Ivoire, though not in Tanzania, showed that higher socioeconomic status and earning own income were significant predictors of willingness to use HIVSTK in unadjusted models. The lack of association between resource and willingness to use HIVSTK is consistent with evidence from Kenya where among ANC and postpartum clients, socioeconomic status did not predict willingness to use HIVST [15]. Women in the Kenya study were willing to pay a similarly low price of about $1 USD, and the authors assert that this lack of statistical significance of economic factors on willingness to use HIVST indicates pricing must stay low or consumers will not be willing to pay for HIVSTK.

A small proportion of women in our study had health insurance, and although having insurance was a predictor for being willing to use an HIVSTK in the Cote d’Ivoire bivariate model, the generally low access to health insurance in both countries is likely to have little impact on uptake of HIVST. Nevertheless, promoting mandating that health insurance coverage include HIVSTK may be one component in a set of national strategies for increasing access.

More economic resources or access to health insurance may facilitate health seeking from the private sector, but only if people perceive the private sector to be providing quality services/products. Our findings on the advantages and disadvantages of using the test kits described in this study highlight the practical, structural and cultural complexities that HIVST policies must consider in order to support high HIVST acceptance. Because the HIV testing infrastructure has been established as a free service, conducted by medical professionals, and under government guidelines, any new modality for HIV self‐testing through the private sector (and largely through pharmacies) will require shifting attitudes and building trust in the quality of those services. For example, one of the main disadvantages women in our study said was the lack of psychological and HIV counselling support if self‐testing. This may be addressed by providing support and links to care through a call‐in number or adding HIVST information and counselling support to existing hotline numbers.. In Nigeria, a call‐in number was provided to recipients of HIVSTK, half of participants who tested HIV positive called in for additional support [9]. Privacy and confidentiality were seen as an advantage of self‐testing and evidence from other studies shows male partners of ANC clients would also value HIV testing modalities that afford greater privacy and confidentiality [23].

Private pharmacies work in informal coordination with public facilities by providing convenient, though with a cost, access to medicines prescribed by clinicians at public facilities, where those medicines are often out of stock in that facility's pharmacy. In addition, they play a role in facilitating access to over‐the‐counter medicine, often purchased in lieu of a clinical/health facility visit where clients perceive advantages regarding opportunity costs (time, travel), and they provide access to elective self‐care products such as condoms and health monitoring kits (e.g. high blood sugar). The majority of private pharmacy respondents reported interest in incorporating HIVSTK into their services. In Cote d’Ivoire, only 73% of pharmacists had ever heard of HIVSTK; however, acceptability of the approach was high, with over 90% reporting interest in stocking them in the future. In Tanzania, 56% of pharmacists knew about HIVSTK and 78% would want to stock them.

The main challenges for pharmacists are similar to other essential commodities that are newly introduced and include stock outs, transport, supply and cost or pricing challenges. Engaging with the private sector could greatly expand the locations where HIVSTK are made available. This would require some investment in training private sector pharmacy staff in the reporting systems used in the public sector for HIV testing services, as well as ensuring adherence to other health systems regulatory requirements. Pharmacists will require training and guidance in offering HIVSTK, and potentially building pharmacy staff skills in providing pre‐ and post‐test counselling and links to confirmatory testing, care and treatment services, and/or establishing call‐in numbers available to access counselling or help troubleshoot testing challenges, as well as linking callers to care. Some pharmacies may lack the physical space for individuals to receive confidential counselling, and this should be a consideration in ensuring quality of services provided. Therefore, a coordinated health systems approach engaging public and private sectors to strengthen supply chains, establishing acceptable pricing, providing training for HIV counselling and testing and HIV reporting systems, guidance in offering HIVST, and coordinating linkages to care and follow‐up systems is required.

There are several limitations to this study. HIVSTK are not yet available in Cote d’Ivoire or Tanzania, thus none of the study participants had actual experience with HIVSTK. Views on HIVSTK, advantages and disadvantages, important qualities and price were all provided in the hypothetical, which may not be an accurate predictor of future behaviour. This was mitigated by interviewers using a standardised script explaining what an oral HIVSTK is and how it is used.

The study was not designed to assess male partners’ perspectives and views on HIVSTK, or if they would use an HIVSTK shared with them by their female partner. Some gendered differences exist on motivations for men in HIV testing in the context of couples [23, 24] and would be important to consider in planning for self‐testing. The study was also limited by use of convenience sampling and a small sample of high‐volume facilities in urban areas. Use of convenience sampling may have introduced bias in the recruitment of ANC clients, leading to less reliable estimates of willingness to use HIVSTK in the future. These results are not generalisable to other urban areas in sub‐Saharan Africa..

## Conclusions

HIVSTK were found to be highly acceptable among ANC clients in Cote d’Ivoire and Tanzania, with the price that women are willing to pay for them similar to that for pregnancy self‐test kits. Providing HIVSTK at low cost will likely ensure more consumers will use them. Consideration should be given to providing psychological and counselling support, and ensuring linkage to HIV services for those testing positive. Engaging the private sector, including pharmacies, will be an important strategy to optimise implementation of an HIV self‐testing program.

The governments of Cote d’Ivoire and Tanzania are currently exploring how to integrate HIVSTK into their policies. These findings will be useful in the formulation of other sub‐Saharan African national HIVSTK policies and to inform national planning for introduction and roll‐out in order to strategically maximise access to HIVSTK for high‐risk populations.
